# Risk Factors and Long‐Term Outcomes in Horses After the 2021 Outbreak of Equine Herpesvirus 1 Myeloencephalopathy, Valencia, Spain

**DOI:** 10.1111/jvim.70040

**Published:** 2025-03-07

**Authors:** María de la Cuesta‐Torrado, Ana Velloso Alvarez, Isabel Santiago‐Llorente, Lara Armengou, Federico Nieto, José Ríos, Fátima Cruz‐López, Eduard Jose‐Cunilleras

**Affiliations:** ^1^ Dept. Animal Medicine and Surgery Universidad Cardenal Herrera‐CEU, CEU Universities Alfara del Patriarca Spain; ^2^ Hospital Clínico Veterinario Complutense Universidad Complutense Madrid Spain; ^3^ Unitat Equina Fundació Hospital Clínic Veterinari Barcelona Spain; ^4^ Equihealth Veterinarios Barcelona Spain; ^5^ Department of Clinical Farmacology, Hospital Clinic and Medical Statistics Core Facility, Institut d'Investigacions Biomèdiques August Pi i Sunyer (IDIBAPS), 08036 Barcelona, Spain; and Biostatistics Unit, School of Medicine Universitat Autònoma de Barcelona Barcelona Spain; ^6^ VISAVET Health Surveillance Centre Universidad Complutense Madrid Spain; ^7^ Dept. Animal Medicine and Surgery Universitat Autònoma de Barcelona Barcelona Spain

**Keywords:** EHV‐1, horse, quarantine, risk factor, vaccine

## Abstract

**Background:**

Equine herpesvirus myeloencephalopathy (EHM) is a persistent threat to horses, with unclear risk factors and disease severity.

**Objectives:**

To evaluate risk factors, effective reproduction rate (Rt), and long‐term athletic outcomes of an EHM outbreak.

**Methods:**

Retrospective study of the 2021 EHM outbreak in Valencia, Spain, examining associations between risk factors (sex, age, breed, country of origin, and vaccination status) and case fatality rate, EHM development, and odds of returning to competition using odds ratios [95% CI] and Rt via the Robert Kochs Institute method.

**Results:**

Among 191 horses, 38 (20%) were clinically normal, 13 (7%) were subclinical, and 140 (73%) presented clinical signs (89 EHM, 64%). One hundred sixty horses were isolated at the show, while 47 were treated in hospitals. The mean age was 9.8 ± 3.0 years; 85 (45%) were mares, 79 (41%) geldings, and 27 (14%) stallions. The EHM case fatality rate was 11/89 (12%). Vaccination was associated with EHM development (4.54[2.23–9.27]; OR[95% CI]; *p* < 0.001) and case fatality rate (3.9[1.1–14.4]; OR[95% CI]; *p* < 0.043). EHV‐1‐infected horses without EHM were more likely to return to competition (54/61; 89%) than those recovering from EHM (65/89; 73%; *p* = 0.024). It was initially 4.2 and decreased to < 1 within 2 weeks of the outbreak.

**Conclusions:**

During the 2021 EHV‐1 outbreak in Valencia, vaccination status appears to be associated with EHM development. Horses recovering from EHM had slightly lower chances of returning to competition than those shedding EHV‐1 without EHM. The high R_t_ value underscores the contagious nature of EHV‐1.

**Abbreviations**
BWP: Belgian Warmblood
CNS: central nervous system
EHM: equine herpesvirus myeloencephalopathy
EHV: equine herpesvirus
FEI: International Equestrian Federation
MLST: multilocus sequence typing
R: reproduction number
R_0_
: basic reproduction number
RoW: rest of the world
R_t_
: effective reproduction number
VTH: veterinary teaching hospital

## 1 Introduction

Equine herpesvirus type 1 (EHV‐1) is a highly contagious virus affecting horses worldwide [1, 2]. The most characteristic clinical signs are mild respiratory signs with fever in horses less than two years old, abortions during the third trimester in pregnant mares, early neonatal deaths, and outbreaks of equine herpesvirus myeloencephalopathy (EHM) [3, 4, 5]. EHV‐1 infections are relatively common in young horses, resulting in the establishment of latent infection within the first few weeks or months of life [3, 4]. The estimated prevalence of latent infection ranges between 54%–88% [5]. During periods of stress, viral reactivation can trigger clinical disease and viral shedding, making the surveillance of EHV‐1 infections challenging. The development of EHM, which is considered a complication of EHV‐1 respiratory tract infections and viremia, is highly complex [6, 7]. During an EHV‐1 outbreak, approximately 10% of infected horses develop EHM; this complication is associated with a poor prognosis [8].

In the last few decades, the incidence of EHM outbreaks has increased worldwide [3, 4, 6]. Equestrian sports activities are commonly associated with a high prevalence of risk factors [9, 10, 11], such as high viral load, host characteristics, and favorable environmental conditions that can all play significant roles in modulating individual and collective clinical signs [1, 8, 12, 13, 14, 15]. Although many epidemiological factors are still under investigation [16, 17, 18], the information in the literature regarding long‐term athletic outcomes in horses affected by an EHV‐1 outbreak is limited.

In February 2021, the Valencian Community (Spain) experienced a severe outbreak of EHV‐1 caused by the N variant (point mutation EHV‐1 A2254) virus strain during an international equestrian sporting event [19]. This outbreak had serious repercussions for the equine sector due to the large number of affected horses and its rapid worldwide spread, resulting in a substantial international economic impact. The study of this outbreak provided valuable data to expand the available knowledge on key factors influencing the development of EHM and the likelihood of horses affected by an EHV‐1 outbreak returning to exercise. Additionally, new aspects that had not been previously studied, such as the reproduction number (R_0_), were also investigated.

The basic reproduction number (R_0_) is defined as a value that indicates the average number of secondary cases of a disease that can be generated from a single primary case in a susceptible population, under ideal conditions, meaning without interventions or control measures. R_0_ can be interpreted as the likelihood that the introduction of an infectious individual into a susceptible population will result in a significant outbreak. This variable is a reliable estimator of the severity of an outbreak and allows the quantification of the transmission potential of a disease [20]. Furthermore, it can be used to assess the changes in infection dynamics as part of the population becomes vaccinated or as the level of immunity changes as an outbreak progresses [21]. Currently, there are no reports on the R_0_ values of EHV‐1 infections. Therefore, the present study aimed to describe the epidemiological characteristics of the 2021 EHV‐1 Valencia outbreak [22]. The objectives of this study were to 1) evaluate the risk factors associated with the development of EHM or fatality rate, 2) estimate the effective reproduction number (R_t_), and 3) evaluate the long‐term outcomes of affected horses based on the level of performance 30 months after infection.

## 2 Materials and Methods

This epidemiological study involved the analysis of retrospectively collected data of horses from an EHV‐1 outbreak that occurred in Valencia, Spain, in February 2021 during a show jumping International Equestrian Federation (FEI) competition event [22, 23]. Clinical records were obtained from the veterinary team at either the competition site or from three referral equine hospitals that treated the affected animals: the three Veterinary Teaching Hospitals (VTH) at the Universities of Madrid, Barcelona, and Valencia, Spain. Owing to the high number of horses present at sports competition facilities, the horses were housed in 440 portable stalls located within a single large temporary tent (Figures S1 and S2).

### 2.1 Description of the Outbreak

On February 14th, four horses out of a group of 16 that left the facilities became pyrexic (rectal temperature > 38.3°C). Upon reaching the destination in France on February 16th, the horses tested positive for EHV‐1. On February 20th, the first horse was referred from the CES Valencia Spring Tour to the Valencia Veterinary Teaching Hospital, presenting with fever and ataxia. On the same day, the FEI Veterinary Department was informed of 20 febrile horses at the event and a confirmed case of EHV‐1 neurological disease in a horse that had recently returned to France after competing in the CES Valencia Spring Tour. As a result, the four‐week jumping tour, which was set to feature 752 competing horses, was canceled. Ten different National Federations (NFs) reported cases epidemiologically linked to the CES Valencia Spring Tour outbreak [22].

### 2.2 Collection of Clinical and Epidemiological Data

Horses isolated during the CES Valencia Spring Tour outbreak (*n* = 160) and those who had direct contact and were admitted to one of the three referral hospitals (*n* = 31) were evaluated. The horses were referred to hospitals based on the severity of their signs of neurologic disease. Most horses were referred directly from the competition site, although some had already returned to the home stable when clinical signs emerged and were therefore referred from those actual locations. Neurological examinations were performed by competition and hospital veterinarians, and horses were assessed daily by evaluating their mental status, cranial nerve function, gait analysis while walking outside the stall, and assessment of tail and anal tone, as well as the onset of fever. Fever was defined as a rectal temperature above > 38.3°C. Neurological deficits were assessed using a modified scale developed by de Lahunta and Mayhew [24]. Horses that showed an ataxia grade ≥ 3/5, altered mental status, cranial nerve dysfunction, or some combination of these signs, had priority for referral. In addition, horses with lower‐grade ataxia (0–2/5) that showed difficulty in urination resulting in the need for long‐term urinary catheterization, or colic signs were also included in the admission criteria. Horses displaying clinical signs, that did not meet the inclusion criteria previously described for hospital referral or for which access to the VTH was not possible owing to the rapid progression of signs of neurologic disease, were treated on‐site by a team of 13 national and international veterinarians. The recorded data included the sex, age, breed, country of origin, presence of signs of neurologic disease (mental status alteration/ataxia/urinary incontinence), vaccination status for EHV‐1, treatment received, and onset of fever. Full physical examination was performed at least twice a day at the venue, and 6 times a day at the hospital. Horses that did not present with fever, neurological or other clinical signs, and tested negative by PCR on three separate dates were considered as ‘clinically normal horses’. Horses that did not develop any clinical signs but for which one of the analyzed samples tested positive by PCR were considered as ‘subclinically infected horses’. Short‐ and long‐term outcomes in terms of fatality rate and the likelihood of return to competition were studied. Performance data were reviewed using the FEI horse database. The decision to perform euthanasia was made based on the identification of advanced neurological alterations that were unresponsive to treatment or systemic complications (urinary or vascular) [25] resulting from the disease with poor prognosis, after receiving the owner's informed consent.

### 2.3 EHV‐1 Vaccination Status

The EHV‐1 vaccination records were obtained from the horses' respective passports. Horses vaccinated with 2 first doses at 4‐week intervals, followed by at least one booster yearly, were considered correctly vaccinated. However, those that had not been vaccinated at the time, had no records of vaccination against EHV‐1, or had doubtful data in their passports were considered unvaccinated.

### 2.4 Classification of Horse Breeds and Country

The horses involved in this outbreak belonged to various warmblood breeds. This factor was analyzed in two different ways: first, for direct comparison with recent studies on EHV1/EHM outbreaks, horse breeds were classified according to previously reported phylogenetic lines [26]. Second, horses of the same breed with ≥ 14 members were grouped together, while the rest of the horses were classified into a group called “others”. Breed classification was: Belgian Warmblood (BWP; *n* = 19), Hanoverian (*n* = 14), Holsteiner (*n* = 16), Koninklijk Warmbloed Paardenstamboek Nederland (KWPN; *n* = 24), Oldenburg (*n* = 19), Selle François (*n* = 37), and Zangersheide (*n* = 15). The rest of the horses (*n* = 47) belonged to 11 other breeds and were grouped together. When horses were grouped into main genetic lines to analyze the data, as performed in previous studies [27, 28], all horses except two were classified in line 1 (warmblood breeds); of the remaining two horses, one (Connemara) was classified in line 5 (British Isles pony breeds), and the other (Canadian sport horse) was considered a crossbreed between two clusters and was excluded from the breed analysis.

For the country of origin analysis, groups with ≥ 10 members were classified independently, while the rest were grouped as “rest of the world” (RoW), except for adjacent geographical grouping (e.g., Portugal and Spain). This criteria yielded 9 categories: (1; *n* = 34) Spain (33) and Portugal (1); (2) France (*n* = 23); (3) Belgium (*n* = 18); (4) Netherlands (*n* = 12); (5) Germany (*n* = 17); (6) Sweden (*n* = 24); (7) Denmark (*n* = 12); (8; *n* = 20): Ireland (8) and United Kingdom (12), and rest of the word (RoW; *n* = 21) Canada (3), China (4), Finland (1), Hong Kong (4), Republic of South Africa (8), and Russia (1). The country of origin could not be identified in 10 horses.

### 2.5 Diagnosis of EHV‐1 Affected Horses

Disease diagnosis was based on a positive EHV‐1 PCR result from at least one of the nasopharyngeal swabs or blood samples in EDTA tubes taken during competition or at the hospital. Samples were analyzed at two national official laboratories in Spain: the National Reference Laboratory (Algete, Spain) and the VISAVET Health Surveillance Centre (Madrid, Spain). Regardless of the results, all horses admitted with fever or neurological alterations were treated and isolated as suspected of being infected until the next PCR was performed due to origin from the outbreak location. Horses were considered positive only if at least one test yielded a positive result.

### 2.6 Follow‐Up of Return to Athletic Activity

Data for each horse were collected from the FEI website (https://data.fei.org/horse). Those that re‐entered the FEI competitions approximately 30 months after the outbreak were considered fit to return to exercise. The horses were classified into two groups: returned and not returned to the FEI jumping competition.

### 2.7 Estimation of Effective Reproduction Number and Spread of Cases During the Outbreak

The R_t_ during the exponential epidemic growth was estimated using the Robert Kochs Institute method [29]. This variable is defined as the expected number of new infections caused by an infected individual in a population, where some individuals may no longer be naïve. This probability is expressed as 1–1/R_t_, provided that R_t_ > 1 and the duration of the infection follows an exponential distribution. For this study, the time from infection to the development of fever was estimated to be 4 days, based on previously reported data on experimental EHV‐1 infection in adult horses [30, 31].

Spatial and temporal analyses of new cases during the outbreak in the competition grounds were performed by calculating the proportion of horses diagnosed with EHV‐1 infection over a 7‐day period and segmenting data over blocks of 20 adjacent stables. Data are presented as the relative number of new cases over a 7‐day period, with a range of 0–1 and intervals of 0.1, with 0 being the minimum and 1 being the maximum values (i.e., either none or all horses in that group were non‐infected or new cases).

### 2.8 Statistical Analyses

Risk factors, including sex, age, breed, country of origin, vaccination, and treatment, were investigated for the association with three outcomes of interest: (i) development of EHM, (ii) death, and (iii) return to competition within 30 months, by calculating the odds ratios (OR) and 95% confidence intervals [95% CI]. Age was analyzed both as a continuous and as a categorical variable, classifying horses into 4 quartiles (≤ 7, 8–9, 10–11, ≥ 12). In addition, the country of origin (i.e., federation) of the horses was investigated for its association with the development of EHM. These estimates were derived from univariate logistic regression models. Notably, ORs were not calculated for comparisons involving fever by EHM status or death, or for signs of neurologic disease by death due to the absence of cases in any category. In such instances, Fisher's exact test was applied to assess statistical significance without OR estimation. Susceptibility to confounding factors was addressed using multiple logistic regression models, integrating variables such as sex, age, breed, or country of origin and vaccination status as potential risk factors. These models were then applied to examine the association between vaccination status and the development of EHM and mortality. All analyses were performed using commercially available software (IBM SPSS Statistics software version 26, IBM Corp. Armonk, NY. USA), with significance set at a two‐sided p‐value ≤ 0.05.

## 3 Results

### 3.1 Description of Cases

**FIGURE 1:**
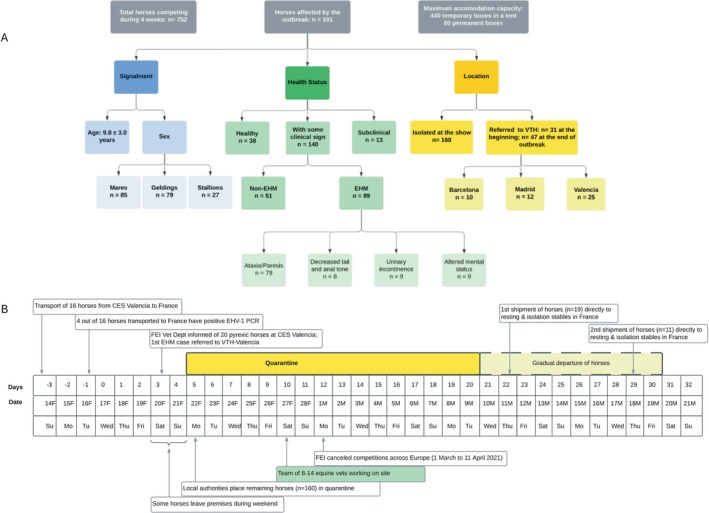
Flow chart of 191 horses included in this study (A), and summarized timeline of dates and events (B) during the EHV‐1 outbreak in Valencia in February 2021. The neurologic signs displayed by horses affected by EHM are detailed independently in the figure; horses could show more than one sign of neurologic disease simultaneously.

A total of 191 horses were included; 160 horses were isolated at the show, and 31 were referred to different VTHs in Spain. These 31 horses had left the competition without apparent clinical signs before declaring quarantine at the venue and had developed clinical signs of EHV‐1 days later (Figure 1).

Mean ± standard deviation age of the horses was 9.8 ± 3.0 years old (range 5 to 21), with most aged between 6 and 13 years of age (167/189; 88%). Regarding sex distribution, 85 (45%) were mares, 79 (41%) were geldings, and 27 (14%) were stallions.

At the end of the outbreak, 47 horses had been treated at the three VTHs (Barcelona, *n* = 10; Madrid, *n* = 12; Valencia, *n* = 25). In total, 38/191 (20%) horses remained clinically normal, 13/191 (7%) were subclinical cases, and 140/191 (73%) presented with at least one clinical sign, including fever (112/140, 80%), EHM (89/140, 64%), and non‐pruritic wheals or lower limb edema (5/140, 4%). The signs of neurologic disease observed included hind limb ataxia and paresis (79/89, 88%), decreased tail and anal tone (8/89; 9%), and urinary incontinence (15/89, 17%). More severely affected horses displayed an altered mental status with stupor combined with episodes of hyperreactivity (9/89, 10%; Figure 1). Furthermore, 25/89 (28%) horses that developed EHM had fever. PCR analysis results were available for 180/191 (94%) horses, of which 86/180 (47%) were negative and 94/180 (52%) were positive (13 of 94 were subclinical cases, PCR‐positives). The remaining 11/191 (5%) horses could not be tested due to insufficient volume of whole blood collected, or the results were inconclusive, indicating a non‐sigmoidal amplification curve below the established threshold.

Vaccination status was verified in 175 out of 191 horses (92%); the remaining animals, 16/191 (8%), could not be classified as vaccinated or non‐vaccinated as this information was unavailable. Then, 52/175 (30%) horses had been vaccinated against EHV‐1, while 123/175 (70%) had not been vaccinated. No statistically significant differences were observed in horses vaccinated against EHV‐1 when grouped by sex: 29/79 (37%) mares and 23/96 (24%) geldings and stallions (*p* = 0.07).

### 3.2 Association Between Risk Factors and Development of EHM and Fatality Rate

Prevalence of EHV‐1 infection, based on the presence of clinical signs or PCR detection, was 153/191 (80%) horses. The case fatality rate of EHM was 11/89 (12%). All deaths were related to the unfavorable progression of EHM. There was no significant difference (*p* = 0.14) in the case fatality rate with respect to age or sex, but mares were overrepresented among non‐surviving horses (8/11 [72%], 3/11 [27%], and 0/11 [0%] for mares, geldings, and stallions, respectively; Table 2). Regarding the breeds, relative to BWP, KWPN showed a lower likelihood of developing EHM signs (0.30 [0.09; 1.02]; *p* = 0.053), while Oldenburg had a higher likelihood of developing EHM signs (3.64 [0.91;14.61]; *p* = 0.069). Grouping the breeds by genetic line did not result in a classification that would allow for analysis. The analysis of the country of origin demonstrated that relative to Belgium, the Netherlands (0.03[0.00; 0.35]; *p* = 0.004), Sweden (0.16[0.04;0.61]; *p* = 0.008), and the RoW (0.24[0.06;0.92]; *p* = 0.037) had a lower likelihood of developing EHM, but no differences were found between other countries.

Vaccination status was significantly associated with the development of neurological deficits; 14/91 (15%) non‐neurologic horses were vaccinated, whereas 38/84 (45%) EHM horses were vaccinated. Of the 123 unvaccinated horses, 77 did not present with signs of neurologic disease, while 46 presented with signs of neurologic disease (4.54 [2.23–9.27]; OR [95% CI]; *p* < 0.001; Table 1). Furthermore, vaccination was significantly associated with case fatality rate: 46 of the 165 survivor horses were vaccinated; in contrast, 6 of the 10 non‐survivor horses were vaccinated (3.9 [1.1–14.4]; OR [95% CI]; *p *= 0.04; Table 2). The association of age with the development of EHM was not significant when age was considered as a linear relationship; however, when age was considered as a possible risk factor according to the four main age groups, an association with EHM was observed in horses aged 8–11 years relative to younger horses (*p* = 0.00; Table 1). In cases of EHM, vaccination status maintained statistical significance with an OR of 4.4 [2.2–9.1] (*p* < 0.001) when adjusted for sex (OR 1.3 [0.7–2.4]; *p* = 0.49). Similarly, vaccination status maintained statistical significance when adjusted for age as 4 categories, with an OR of 4.1 [1.9; 8.5] (*p* < 0.001). Regarding the association between vaccination and case fatality rate, there was no statistical significance, with vaccinated horses having a crude OR of 3.3 [0.9–12.4] (*p* = 0.08) relative to non‐vaccinated animals, and similarly when adjusted for sex (OR 4.6 [0.9–22.7]; *p* = 0.06). Finally, vaccination was also proved significant when adjusted for breed and country, with an OR of 4.7 [2.2–10.1] (*p* < 0.001) and of 4.0 [1.7–9.1] (*p* < 0.001), respectively.

**TABLE 1:** Association of risk factors with EHM development.

Risk factor	Categories/Units		Non‐EHM	EHM	OR (95% CI)	*p*
Age	Years	Mean (SD)	9.8 (3.4)	9.8 (2.5)	1.00 (0.91;1.10)	0.98
		Median [P25th, P75th]	9 [7;11]	9 [8;11]		
		N (Range)	100 (5–21)	89 (5–16)		
		< 7y	34 (34%)	14 (15.73%)	[Ref]	
		8–9 y	20 (20%)	31 (34.83%)	3.76 (1.63; 8.71)	0.00
		10–11y	16 (16%)	24 (26.97%)	3.64 (1.50; 8.85)	0.00
		> 11 y	30 (30%)	20 (22.47%)	1.62 (0.70; 3.75)	0.26
Sex	Stallion	N (%)	16 (15.69%)	11 (12.36%)	[Ref]	
	Mare	N (%)	41 (40.20%)	43 (48.31%)	1.53 (0.63; 3.67)	0.34
	Gelding	N (%)	45 (44.12%)	35 (39.33%)	1.13 (0.47; 2.74)	0.78
	Mare vs other	N (%)			1.39 (0.78; 2.47)	0.2
Vaccination	No	N (%)	77 (84.62%)	46 (54.76%)		
	Yes	N (%)	14 (15.38%)	38 (45.24%)	4.54 (2.23; 9.27)	< 0.001
	Vaccination adjusted by age (categorical)				4.06 (1.94; 8.51)	< 0.001
	Vaccination adjusted by sex				4.41 (2.15; 9.05)	< 0.001
	Vaccination adjusted by breed				4.69 (2.18;10.09)	< 0.001
	Vaccination adjusted by country				3.99 (1.73–9.19)	0.001
Fever	No	N (%)	51 (50%)	0 (0%)		
	Yes	N (%)	51 (50%)	89 (100%)	NE	< 0.001
Maximal Temperature	°C	Mean (SD)	38.0 (0.60)	38.4 (0.75)	2.49 (1.34; 4.65)	0.00
		Median [P25th, P75th]	37.80 [37.70; 37.70]	38.25 [37.85; 37.85]		
		N (Range)	45 (37.1–40.0)	76 (36.0–40.0)		

Abbreviations: NE, Not Evaluated Because of Zero Values in Cells; [Ref], Reference Category.

**TABLE 2:** Association of explanatory variables with case fatality rate.

Risk factor	Categories/Units		Survivors	Non‐survivors	OR (95% CI)	*p*
Age	Years	Mean (SD)	9.7 (3.0)	11.3 (2.9)	1.16 (0.97;1.39)	0.09
		Median [P25th, P75th]	9 [7;11]	11 [9;13]		
		N (Range)	178 (5–21)	11 (6–15)		
		< 7y	47 (26.40%)	1 (9.09%)	[Ref]	
		8–9 y	49 (27.53%)	2 (18.18%)	1.92 (0.17;21.87)	0.6
		10–11y	37 (20.79%)	3 (27.27%)	3.81 (0.38;38.15)	0.25
		> 11 y	45 (25.28%)	5 (45.45%)	5.22 (0.59;46.46)	0.13
Sex	Stallion	N (%)	27 (15.0%)	0 (0.0%)	[Ref]	0.14
	Mare	N (%)	76 (42.2%)	8 (72.7%)		
	Gelding	N (%)	77 (42.8%)	3 (27.3%)		
	Mare vs other	N (%)			3.65 (0.94;14.2)	0.06
Vaccination	No	N (%)	119 (72.1%)	4 (40%)		
	Yes	N (%)	46 (27.9%)	6 (60%)	3.88 (1.05;14.38)	0.04
Fever	No	N (%)	51 (28.3%)	0 (0%)		
	Yes	N (%)	129 (71.7%)	11 (100%)	NE	0.03
Maximal Temperature	°C	Mean (SD)	38.3 (0.67)	38.1 (1.2)	0.72 (0.27; 1.96)	0.52
		Median [P25th, P75th]	38.0 [37.8; 37.8]	37.8 [37.6; 37.,6]		
		N (Range)	112 (37.1–40.0)	9 (36.0–40.0)		
Neurologic signs	No	N (%)	102 (56.7%)	0 (0%)		
	Yes	N (%)	78 (43.3%)	11 (100%)	NE	< 0.001
Grade of ataxia	0 to 5	Median [P25th, P75th]	0.0 [0.0; 0.0]	5.0 [5.0; 5.0]	8.3 (2.6; 26.6)	< 0.001
		N (Range)	171 (0–5)	10 (2–5)		

### 3.3 Progression of the Effective Reproduction Number and Spread of Cases During the Outbreak Over Time

**FIGURE 2:**
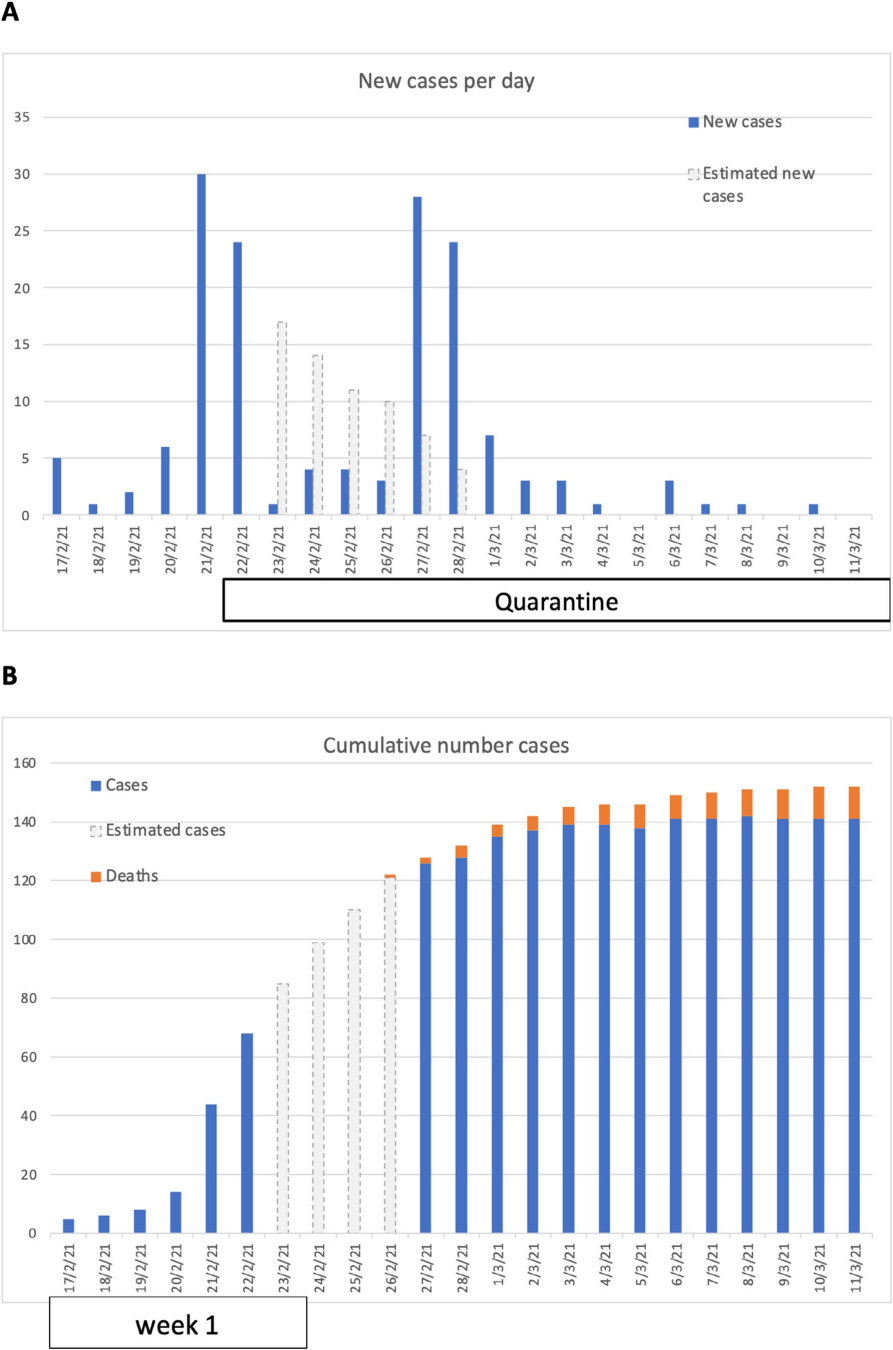
Epidemic growth curve of new cases (A) and the cumulative number of cases (B). Blue depicts suspected EHV‐1 infected cases, as noted in clinical records; orange denotes fatalities, and dashed gray represents the estimated cases during five days without written records.

The estimated R_t_ during the first week of the outbreak was 4.2 (Figure 2), decreasing rapidly to < 1 thereafter. Detailed information on the housing of the horses during the competition is described in (Figures S1 and S2).

### 3.4 Therapeutic Management of Affected Horses

The therapeutic management of EHM‐affected horses was tailored to address individual horse requirements. Stall confinement was established for at least the 1st week after infection. Treatment was adapted according to the recommendations of the EHV‐1 consensus statement, based on the daily neurological examinations [9]. A detailed description of the treatment of horses is included in the (Figure S2).

### 3.5 Follow‐Up of Return to FEI Competition

Out of 191 animals, 18% (35) did not compete again at the FEI level over the next 30 months after the outbreak, while 79% (151 out of 191) did compete again (Table 3). The remaining 5/191 (3%) horses were not show jumpers before or after the EHV‐1 outbreak. EHV‐1 infected horses that did not develop EHM were more likely to return to competition (54/61, 89%) than those that recovered from EHM (65/89, 73%; *p* = 0.024). Further, the ataxia grade presented a negative impact, as horses with lower grades of ataxia were more likely to compete again compared with horses with higher grades (Table 3). No associations were observed between sex, age, vaccination status, or return to the FEI jumping competition.

**TABLE 3:** Association of explanatory variables with return to FEI jumping competition by 30 months.

Variables	Categories/Units		Retired from competition	Returned to competition	OR (95% CI)	*p*
Age	Years	Mean (SD)	10.3 (3.04)	9.7 (3.0)	0.94 (0.83; 1.06)	0.28
		Median [P25th, P75th]	10.0 [8.0; 12.0]	9.0 [7.0; 11.0]		
		N (Range)	35 (5.0–17.0)	151 (5.0–21.0)		
		< 7y	7 (20%)	38 (25.17%)	[Ref]	
		8–9 y	8 (22.86%)	43 (28.48%)	0.99 (0.33;2.99)	0.98
		10–11y	7 (20%)	33 (21.85%)	0.87 (0.28;2.73)	0.80
		> 11 y	13 (37.14%)	37 (24.50%)	0.52 (0.19;1.46)	0.21
Sex	Stallion	N (%)	4 (11.4%)	23 (15.2%)	Ref	
	Mare	N (%)	18 (51.4%)	65 (43.1%)	0.63 (0.19; 2.05)	0.44
	Gelding	N (%)	13 (37.1%)	63 (41.7%)	0.84 (0.25; 2.85)	0.78
Vaccination	No	N (%)	21 (65.6%)	99 (70.7%)		
	Yes	N (%)	11 (34.4%)	41 (29.3%)	0.79 (0.35; 1.79)	0.57
Neurologic signs	No	N (%)	11 (31.4%)	86 (57.0%)		
	Yes	N (%)	24 (68.6%)	65 (43.1%)	0.35 (0.16; 0.76)	0.00
Grade of ataxia	0 to 5	Median [P25th, P75th]	2.0 [0.0; 3.0]	0.0 [0.0; 0.0]	0.55 (0.43; 0.70)	< 0.001
		N (Range)	34 (0.0–5.0)	144 (0.0–4.0)		

## 4 Discussion

Previous research has demonstrated that the development of EHM during an EHV‐1 outbreak might be influenced by virus‐dependent, environmental, and host‐related factors [8, 11, 12, 13]. This study highlights the relationship between different individual variables and the association with the development of EHM or increased risk of death after EHV‐1 infection in a severe outbreak.

Sex is an important risk factor for EHM, with most studies indicating that female horses are at a greater risk [9, 11, 28, 32]. Infected female horses are 4.3‐fold more likely to develop EHM than male horses [33]; however, conflicting reports exist [8]. In the present study, males and females showed the same risk of developing EHM. However, 8 out of 11 non‐survivors were mares, which is similar to the findings of Van Maanen et al. in 2011 [12].

Breed might be a factor influencing the development of EHM [15, 26, 28, 32]. In this study, grouping horses by genetic lineage posed challenges in gathering definitive data, as most belonged to genetic cluster group 1 (warmblood breeds). However, when analyzing the breeds independently, KWPN horses showed a lower likelihood, while Oldenburg showed a higher likelihood of developing EHM compared to BWP. Klouth et al. (2022) studied 589 horses across 13 outbreaks, finding that most outbreaks affected a single breed or a specific predominant warm‐blooded breed, with American Quarter Horses accounting for 66% of the total population (genetic cluster 1) [28]. In contrast, none of the outbreaks investigated included only the Arabian, Haflinger, or Icelandic breeds (genetic clusters 2, 4, and 6, respectively). Another retrospective study showed that Standardbreds, Hispanic breeds, and draught breeds were at a higher risk of manifesting EHM in an EHV‐1 outbreak than archetypical ponies [32]. This is consistent with other studies that showed an increased risk of EHM in specific breeds, such as warmblood horses and Fjord horses, as well as a decreased risk in small pony breeds, Shetland, and Welsh ponies [26]. On the other hand, the percentage of horses harboring latent infections appears highly variable and might depend on the geographic region, among other factors [11]. The analysis of the country of origin of this research demonstrated that countries such as the Netherlands and Sweden showed a lower likelihood of developing EHM compared with Belgium. The Netherlands has experienced numerous outbreaks of EHM, which are well‐documented in the scientific literature [32]. In contrast, to the author's knowledge, there is no scientific information documenting substantial outbreaks of the disease in Sweden [11]. Drawing definitive conclusions about this factor in a population of sport horses could be challenging, as these horses frequently move between countries. Therefore, these results should be interpreted with caution, and the effect of country (i.e., federation) was explored as a possible confounding variable for the association of vaccination with EHM.

Immune function and vaccination status have been proposed as possible factors influencing the risk of EHM [11]. In the present study, no significant relationship was found between age and the likelihood of developing signs of neurologic disease or case fatality when age was considered a continuous variable. The mean age for EHM and non‐EHM ill horses was close to 10 years (9.5 ± 2.9 and 10.8 ± 3.4, respectively). However, the fact that there were no animals under 5 years or over 20 years of age could explain this lack of association between age and the development of EHM. Further, age under 3 years appears to be a protective factor [32], while horses over 20 years of age seemed to have a higher predisposition to EHM [13, 34]. In this study, the cohort was divided into four age groups when age was considered a categorical variable, and for this analysis, a significant difference was found in the development of EHM in horses aged between 8 and 11 years, but not in horses over 11 years of age. This result contrasts with the results of other studies [28]. This discrepancy could be due to the type of analysis used, or to the fact that the population of this study comprised sport horses, where 88% were between 6 and 13 years old, which challenges the analysis of this variable. Although age was not a clear determining factor in this study, vaccination appeared to increase the risk of EHM development and was associated with a higher case fatality rate. A potential link between the immunological status, particularly vaccination, and EHM development is suggested in the literature [13]. However, the observed correlation might also be influenced by the higher vaccination rate among older horses, aligning with the established association between EHM and advanced age. In populations where vaccination is uncommon, reports have indicated an increased risk of EHM with age [32]. Nevertheless, horses vaccinated against EHV‐1 within 5 weeks prior to EHV‐1 exposure were more likely to develop EHM than those vaccinated more than 5 weeks prior to exposure [33], suggesting a unique window of vulnerability for recently vaccinated horses. Despite multiple studies questioning the possible association of EHM with vaccinated horses, no clear consensus has yet been reached [26, 33]. A 2023 study by Courouce et al. which included 60 horses of the 2021 Valencia outbreak, concluded that EHV‐1 vaccination did not predispose horses to the development of EHM. However, the present study, which included 191 horses (quarantined on‐site on competition grounds and hospitalized horses), represents a larger sample size than the aforementioned study. On the other hand, a potential limitation could be that the present study could not analyze the timing of the last vaccination for each horse due to the complex situation of the outbreak during data collection. Nevertheless, vaccination reduces or eliminates cell‐associated viremia and stimulates immune responses [35]. Furthermore, a recent meta‐analysis of EHV‐1 vaccination efficacy suggested that modified live vaccines could reduce viremia and nasal shedding, although they might not decrease the risk of developing EHM or the incidence of EHV‐1 abortion [36]. Therefore, the conclusions of this study should be considered when examining vaccinated horses to establish early diagnoses and treatments that could improve their prognosis. However, these findings should not be taken as an indicator to discourage vaccination. The notable decrease in viral shedding observed in vaccinated horses provides a rational basis for administering booster vaccinations to non‐exposed horses at risk of infection. This strategy aims to limit viral shedding in horses exposed to EHV‐1, ultimately lowering the likelihood of individual infection of at‐risk horses [37].

Our assessment indicated that horses that developed EHM were less likely to be re‐registered in an FEI competition than EHV‐1 infected horses without EHM. However, only the return to jumping competition was evaluated, not considering the level of competition or number of wins, as no surveys were conducted with the owners, which is a limitation of the study. A recent study on the same outbreak found that the grade of ataxia and complications associated with EHV‐1 negatively impacted the likelihood of returning to the same performance level [25], but no previous studies have evaluated the long‐term performance of horses after overcoming EHM. However, one study showed that 10/20 horses with mild signs of neurologic disease recovered completely after a year, while only 3/8 horses that showed more severe signs and survived intensive care returned to their previous performance level, with only one reaching the owner's expectations [12]. Given the substantial investment in time and financial resources in these horses, it is crucial for owners of sport horses to make informed decisions based on the chances of complete recovery [5, 38].

This study estimates the Rt for EHV‐1. In epidemiology, the terms reproduction number (R) and effective reproduction number (Rt) are often used interchangeably, although they differ slightly from R_0_. The most correct metric for assessing the current dynamics of infectious disease transmission and the efficacy of control measures is R or Rt [39]. Due to limited data on EHV‐1 in horses, we compared our results with studies on similar variables like R0 and R. Our value of Rt = 4.2 at the most critical moment of the outbreak is comparable to highly contagious human diseases such as rubella (Rt = 6–7) [40], and higher than other equine infectious diseases, such as *Streptococcus equi* spp. or equine influenza (R0 value of 2.0 and R of 2.4, respectively) [41, 42]. Importantly, after the first week of the outbreak, the Rt progressively stabilized below 1. The high initial Rt values for EHV‐1 and equine influenza, unlike *S. equi* , might be due to their ability to spread through aerosols, facilitating efficient transmission over long distances. In the study reporting the influenza outbreak [41], the R value dropped rapidly to 0.1–0.3 once control measures were implemented, pointing to the importance of increased biosecurity as an effective measure against this disease, as well as collective immunization (all animals were vaccinated in this outbreak). Currently, there are no reports in the scientific literature that provide sufficient data to compare the variables studied on biosecurity measurements established during an EHV‐1 outbreak. Insufficient biosafety measures could also introduce bias when assessing the results on the basic reproduction number of this study. Including the basic or effective reproductive number in future EHV‐1 studies might help to identify which biosecurity measurements are most effective in controlling EHV‐1 infections.

Our spatial and temporal analysis of new confirmed cases in this outbreak suggests that the clustering occurred in adjacent stables. This observation agrees with experimental and field studies suggesting that close contact between horses (“nose‐to‐nose contact”) facilitates viral transmission [6]. Therefore, improved biosecurity measures in temporary stabling facilities at competition sites would likely benefit from preventing direct close contact between horses.

The retrospective nature of this study, along with the limited data during the initial 5 days of the outbreak, was an important limitation of this research. Given the high number of severely affected horses and the involvement of several veterinarians in the initial care, this limitation is both understandable and expected in a study of this characteristic. Additionally, there is the possibility of an undetected subclinical EHV‐1 infection circulating within the population before the identification of the first case, which is a recognized phenomenon of EHV‐1 [5, 38] and likely contributes to the spread of some EHM outbreaks. The results of this study might be specific to this outbreak, where a large number of animals from different origins were housed in a single large space and likely stressed by competition and transportation. Despite these limitations, the data provide valuable insights for managing future outbreaks in a similar context.

In conclusion, this study indicates that horses recovering from EHM showed a lower chance of returning to FEI competition compared to EHV‐1‐infected horses without EHM. The high Rt value further indicated the highly contagious nature of EHV‐1 in this environment. Further, this research suggested that vaccination status might be associated with the development of EHM and perhaps an increased case fatality rate. These findings should be considered when examining vaccinated horses during an outbreak and establishing early treatments. However, vaccination should remain a key measure for controlling EHV‐1 in the equine population. Future studies are needed to expand the current knowledge on this topic. Overall, swift biosecurity actions, enhanced surveillance, and strict data logging are imperative for addressing the challenges associated with outbreaks.

## Disclosure

Authors declare no off‐label use of antimicrobials.

## Ethics Statement

All owners of the horses were informed about and accepted the anonymous use of their horses' data for this scientific study. Authors declare human ethics approval was not needed.

## Conflicts of Interest

The authors declare no conflicts of interest.

## Supporting information


Data S1.
