# Erratum for “Risk Factors and Long‐Term Outcomes in Horses After the 2021 Outbreak of Equine Herpesvirus 1 Myeloencephalopathy, Valencia, Spain”

**DOI:** 10.1111/jvim.70084

**Published:** 2025-04-11

M. de la Cuesta‐Torrado , A. V. Alvarez , I. Santiago‐Llorente , et al., “Risk Factors and Long‐Term Outcomes in Horses After the 2021 Outbreak of Equine Herpesvirus 1 Myeloencephalopathy, Valencia, Spain,” Journal of Veterinary Internal Medicine 39 (2025): e70040.40055829 10.1111/jvim.70040PMC11888933

In the above mentioned article, there is an error in the Abstract Results paragraph, last sentence. The sentence should be as follows.

“Rt was initially 4.2 and decreased to < 1 within 2 weeks of the outbreak.”

Also, Reference 29 is incomplete. Reference 29 should be as follows.

“Robert Koch Institud, “Robert Koch Institud [Internet]. Epidemiologisches. Bulletin,” 2020. [cited 2024 Mar 10]. Available from: https://www.rki.de/DE/Aktuelles/Publikationen/Epidemiologisches‐Bulletin/2020/17_20.pdf?__blob=publicationFile&v=1. Accessed: 28 May 2021.”

We apologize for the errors.

