# Supplementary material for: Risk Factors and Long‐Term Outcomes in Horses After the 2021 Outbreak of Equine Herpesvirus 1 Myeloencephalopathy, Valencia, Spain
Source: J Vet Intern Med. 2025 Mar 7;39(2):e70040. doi: 10.1111/jvim.70040 (PMC11888933; doi:10.1111/jvim.70040)
Supplement: Supplementary file 1 — Data S1. [file JVIM-39-e70040-s001.docx]

**Supplementary material**

**S1. Supplementary Figure 1**

Satellite images of the equestrian competition grounds (A and B), map of the site of CES Valencia Spring Tour 2021 (C) and photo of temporary stables inside large tent (D). Temporary stables within large tent where infected horses were kept: marked with red circle (A), with outline of temporary stables (B) and labelled as E7 (C). Permanent stables where non-infected horses were stabled marked with green circle (A) and as E2 (C). Entrance to the competition grounds marked with solid blue arrow. Entrance to each large tent marked with open red arrows. Sources: Google maps (A and B); <https://equipe-online.s3.amazonaws.com/assets/216986/original.png> (C); photo Anna Maxenchs (D).

**
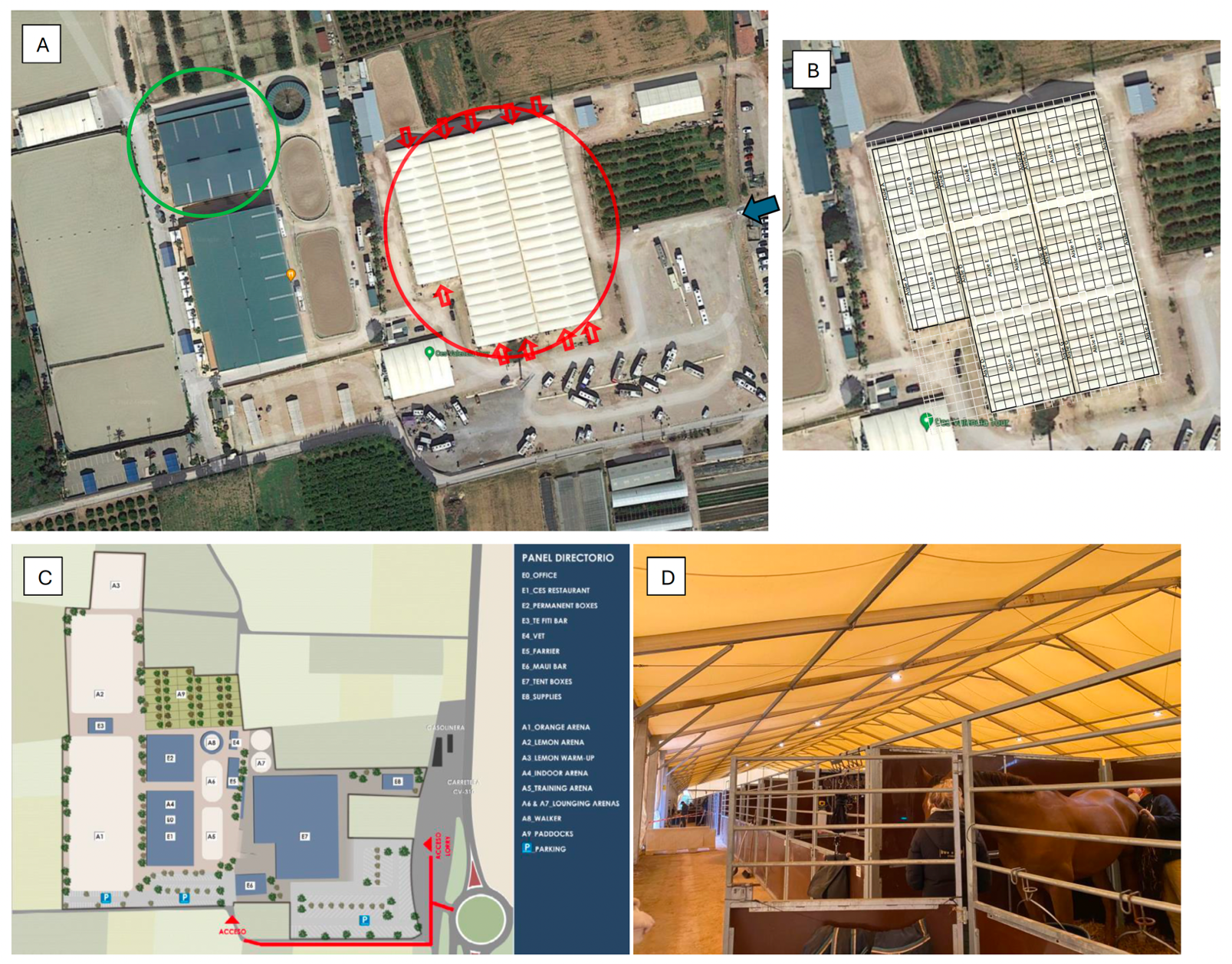
**

**S1. Supplementary Figure 2**

Accumulated incidence over a 7-day period, week one (17 to 23 February 2021), segmenting data over blocks of 20 adjacent stables. Data are presented as relative number of new cases over a 7-day period, with a range of 0 to 1 and intervals of .1, with 0 being the minimum (green) and 1 being the maximum (red) values (i.e. either none or all horses in that group being non-infected or new cases). Intermediate values are shown as shades of light green, yellow and orange with increasing relative number of new cases.

**S2. Overview of the treatments administered during the outbreak in Valencia 2021**

The treatment administered was based on: Flunixin meglumine in 135 (70.7%) of patients (1.1 mg/kg IV q 24h for 3-5 days), dimethyl sulfoxide (DMSO) in 65 (34.0%) (1 g/kg IV q24h for 3 days) and dexamethasone in 84 (44.0%) (0.1-0.2 mg/kg IV q24h for 3 days). Valacyclovir in 38 (19.9%) (30 mg/kg q 8 h PO 7 consecutive days) was administered to symptomatic (fever or neurological signs) horses, with limitations on its prescription for economic reasons or for the possibility of obtaining the medicine, vitamin e was given in 28 (14.7%), heparin in 23 (12.0%) and bladder catheterization for horses with urinary incontinence. Neurologic examination was performed every day a for all horses with the objective of adapting the needs of treatment and management to the progression of each patient. Patients with ataxia grades ≥ 3 were only examined in a straight line outside the box without performing coordination tests on the track. Of neurologically affected horses 9 became recumbent. Therapy included intravenous fluids, tranquilization with valium in case of need, and frequent repositioning or sling support.
